# Anticoagulant and anti-thrombotic therapy in acute type B aortic dissection: when real-life scenarios face the shadows of the evidence-based medicine

**DOI:** 10.1186/s12872-020-01342-2

**Published:** 2020-01-23

**Authors:** Pier Paolo Bocchino, Ovidio De Filippo, Francesco Piroli, Paolo Scacciatella, Massimo Imazio, Fabrizio D’Ascenzo, Gaetano Maria De Ferrari

**Affiliations:** Division of Cardiology, Department of Medical Sciences, ‘Città della Salute e della Scienza’ Hospital, University of Turin, Corso Bramante 88, 10126 Turin, Italy

**Keywords:** Aortic dissection, Coronary artery disease, Intraventricular thrombus, Anticoagulation

## Abstract

**Background:**

Evidence-based recommendations about anticoagulation in acute type B aortic dissection (TBAD) are completely missing, but there is a diffuse conviction that it could prevent the healing process of the dissected aorta’s false lumen. However, several clinical conditions may lead to the necessity to start anticoagulant therapy among patients with acute type B aortic dissection, ranging from atrial fibrillation to more complicated clinical scenarios and the correct management in this kind of patients is still an open issue.

**Case presentation:**

We are presenting a 51-years-old man with multi-infarct encephalopathy referred to us for an acute TBAD and a first diagnosis of ischemic cardiomyopathy complicated by left ventricular (LV) thrombus formation. Coronary angiography revealed a critical stenosis of left anterior descending artery (LAD) treated with drug-eluting stent deployment. The patient was addressed to triple antithrombotic therapy with acetylsalicylic acid, clopidogrel and warfarin with target INR 2.0–2.5. After 6 months, computed tomography angiography revealed the stability of the dissection flap. Cardiac magnetic resonance imaging, however, confirmed the persistence of a small thrombotic formation in LV apex, thus double antithrombotic therapy with warfarin and clopidogrel was instituted. The patient remained asymptomatic during the follow-up period but was advised to suspend his job and physical activities.

**Conclusion:**

Current guidelines do not discuss anticoagulant therapy in the setting of TBAD and large randomized trials are lacking. Despite it is generally considered unsafe to administer anticoagulants in patients with TBAD, we present a case in which triple antithrombotic therapy was well tolerated and did not lead to progression of the intimal flap after 6 months.

## Background

The incidence of acute aortic dissection in the general population is estimated to be about 2.5% per 100,000 person-years [1].

Uncomplicated patients with acute type B aortic dissection are often successfully managed conservatively with a lifelong anti-impulse therapy and serial imaging evaluation, in order to minimize aortic wall stress and to detect extension of the dissection or aneurysm formation respectively. Surgical or endovascular treatment is usually reserved for patients developing complications related to dissection. Even though current guidelines consider patent false lumen as a predictor of aortic growth and poor outcome, there is no mention about the management of antithrombotic or anticoagulant therapy in the setting of aortic dissection [2]. This lack of data is further attested by a relevant interdisciplinary expert consensus on management of type B aortic dissection giving no information about anticoagulation/antiplatelet therapy in this scenario, also due to the large heterogeneity existing among different studies [3]. This substantial gap of evidence compels the physicians to deal with such critical setting without a shared or evidence-based strategy to be pursued.

We report a case of acute TBAD diagnosed along with new-diagnosis of ischemic cardiomyopathy complicated by left ventricular thrombotic formation and patent foramen ovale (PFO) in the setting of multi-infarct encephalopathy. This case outlines the relevance of an individual patient-tailored therapy and further stresses the need of future studies and a consensus about the role of anticoagulant therapy in patients with acute aortic dissection.

## Case presentation

A 51-year-old man was referred to this hospital for a follow-up cardiology visit after a right cerebellar ischemia occurred 1 month prior to presentation, from which the patient had rapidly recovered without residual deficits. He had no history of hypertension, arthritis, uveitis, mouth ulcers nor crystalline lens subluxation. He had been smoking until 2 years before presentation; his anamnesis was negative for alcohol or illicit drug abuse and his family history was unremarkable. His only medication was acetylsalicylic acid.

During the previous hospital stay, extensive examinations had been performed, including electrocardiogram (ECG), lipid panel, transthoracic echocardiography (TTE) and doppler ultrasonographic assessment of the supra-aortic vessels and of the lower extremities’ veins, all of which resulted unremarkable.

A few days after hospital discharge, the patient had suffered a single episode of intense subjective vertigo, tinnitus, confusion and orthostatic imbalance, which had resolved spontaneously in about 20 min without referring to any physician.

On presentation the patient was asymptomatic, eupneic and apyretic. His blood pressure was 155/90 mmHg on both arms. On physical examination a mild pectus excavatum was noticed. Cardiac tones were valid and regular, and a third tone was heard. Radial pulses were symmetrical and valid, while the femoral and pedidial pulses were slightly attenuated.

**Fig. 1:**
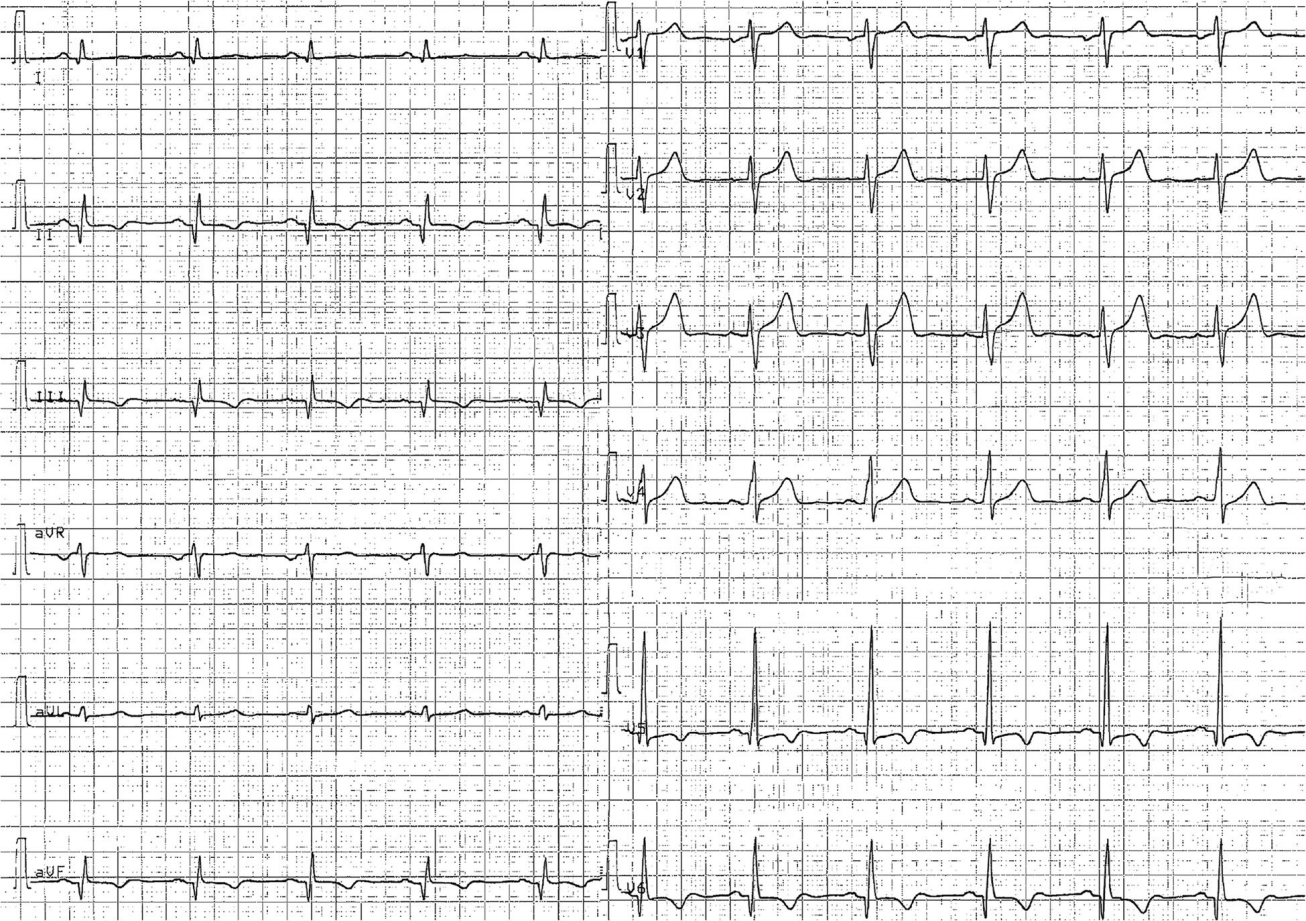
Admission ECG showing Q waves in leads D2, aVF, D3, V5 and V6 and a negative symmetrical T wave in the same leads

The ECG revealed a sinus rhythm at 66 bpm with normal atrioventricular and intraventricular conduction; a Q wave was documented in leads D2, aVF, D3, V5 and V6 and a negative symmetrical T wave was present in the same leads (Fig. 1); these signs were absent in previous ECGs. A TTE was performed, which documented a moderate depression of the left ventricular ejection fraction (LVEF) (39%) and infero-posterior and apical akinesia with left intraventricular “smoke”. The patient was thus admitted to the hospital ward for further investigations.

On admission blood tests were mostly within normal range, including haemoglobin (13,8 g/dL), C-reactive protein (2,4 mg/L), high-sensitivity troponin T measurement (12 ng/L, upper limit of normal 50 ng/L), creatine phosphokinase (90 UI/L), creatinine (0,99 mg/dL), urea (31 mg/dL), aspartate transaminase (16 UI/L), alanine transaminase (12 UI/L), total bilirubin (0,6 mg/dL), INR (1,02), aPTT ratio (0,95), fibrinogen (292 mg/dL). N-terminal prohormone of brain natriuretic peptide was slightly elevated (636 pg/mL, upper limit of normal 300 pg/mL).

**Fig. 2:**
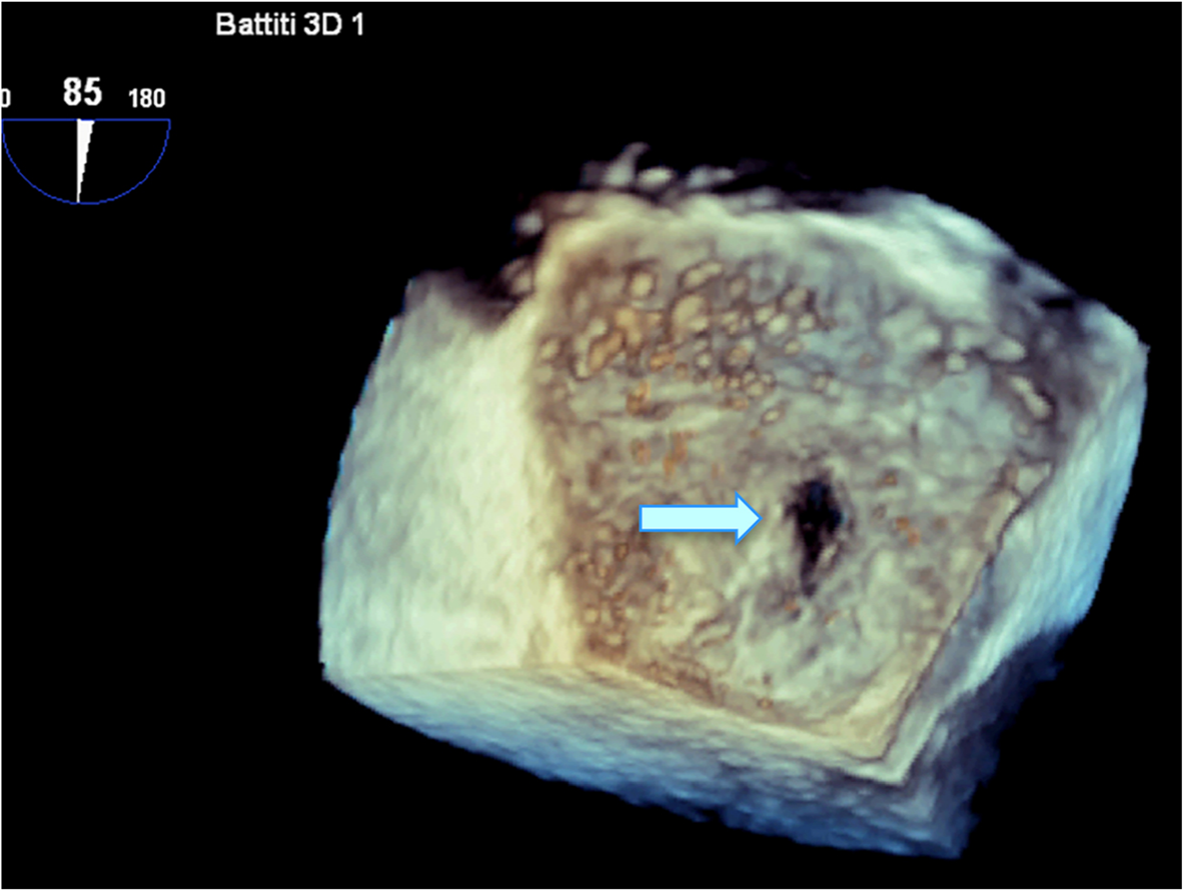
Transesophageal 3D echocardiographic view showing the patent foramen ovale (*light blue arrow*)

**Fig. 3:**
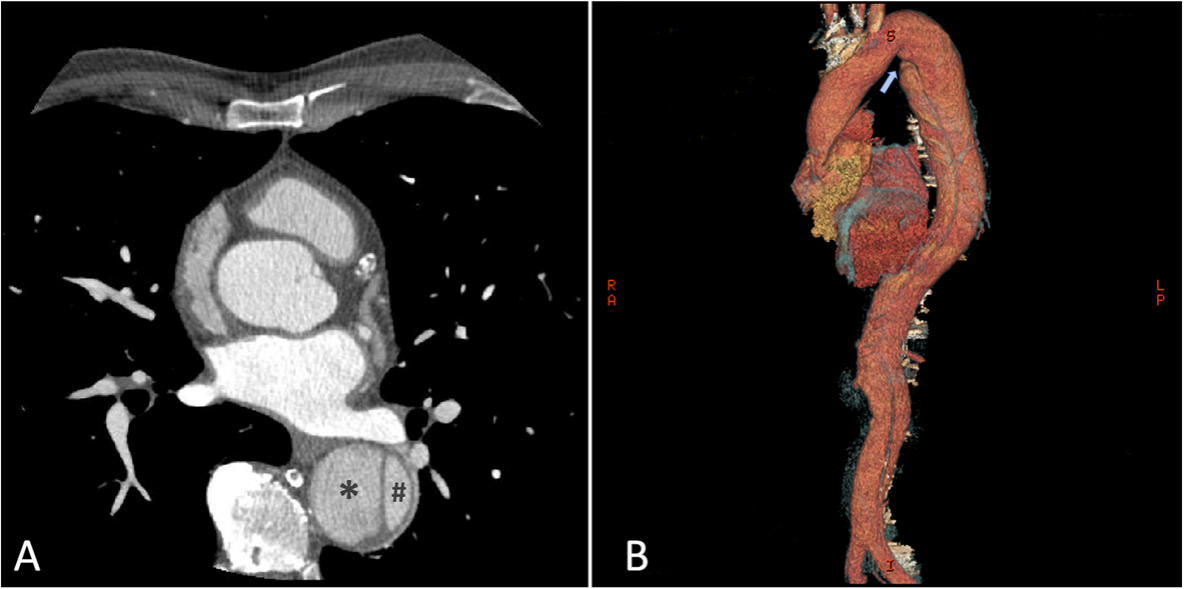
**a** Axial CTA view showing the TBAD with the false lumen (*asterisk*) clearly prevailing over the true lumen (*hash*). **b** CTA 3D-scan documenting the extension of the TBAD, originating from the thoracic descending aorta just below the origin of the left subclavian artery (light blue arrow) to the iliac arteries bilaterally

**Fig. 4:**
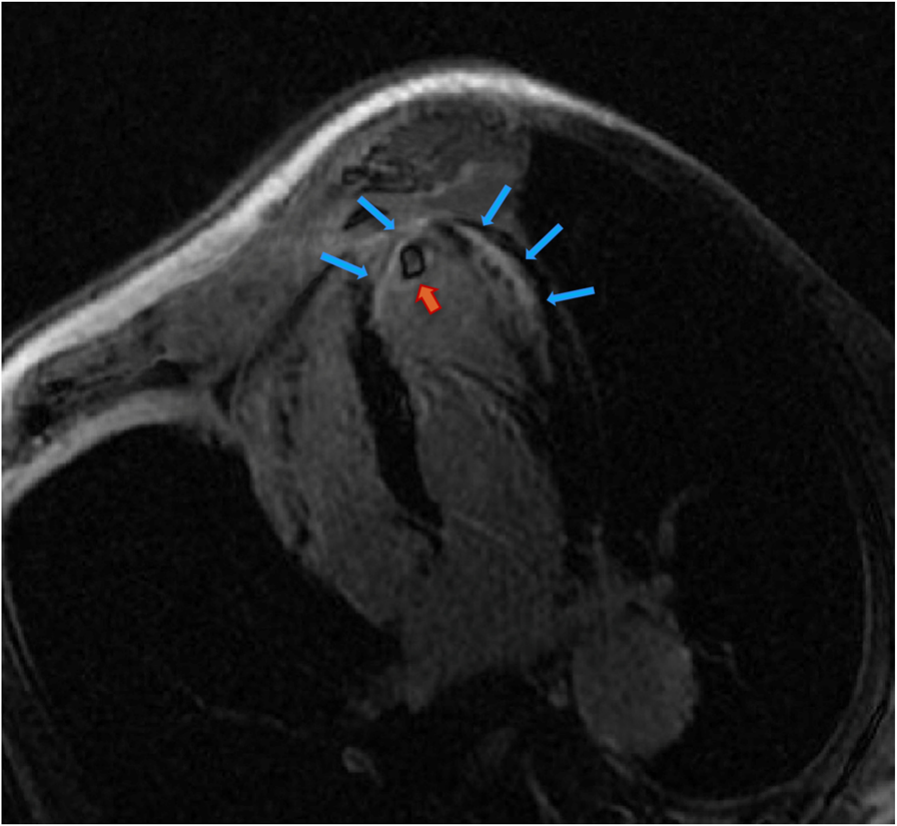
Delayed-enhancement cardiac MR image showing apical intraventricular thrombosis (*orange arrow*) and late gadolinium enhancement (*blue arrows*) in the four-chamber view

Because of the recent stroke, the patient’s rhythm was monitored with ECG telemetry during the whole hospitalization period in order to exclude silent paroxysmal atrial fibrillation episodes. Besides, a transcranial doppler was performed, which documented the presence of a moderate right-to-left shunt. Moreover, the transoesophageal echocardiography (TEE) showed a PFO (Fig. 2) along with a double lumen in the descending thoracic aorta, rising the suspicion of aortic dissection. Consequently a computed tomography angiography (CTA) was performed documenting a TBAD extending from the thoracic descending aorta just below the origin of the left subclavian artery to the iliac arteries bilaterally, the false lumen prevailing over the true lumen (Fig. 3). The right renal artery and the inferior mesenteric artery originated from the false lumen, whereas the left renal artery, the superior mesenteric arteries and the celiac trunk all originated from the true lumen; the supra-aortic trunks and the ascending aorta were not involved in the dissection process. Within the coronary vessels, the CTA documented one-vessel disease with two small eccentric plaques in the proximal left anterior descending (LAD) artery conditioning a moderate stenosis (> 50%). A hypodense area was observed in the subendocardial ventricular myocardium at the apical and middle segments of the inferior wall and at the middle segment of the anterolateral wall; no pericardial effusion was documented. A cardiac magnetic resonance (MR) was then carried out, which showed the presence of ischemic apical, apical-anterior and inferior late-gadolinium enhancement along with intraventricular thrombosis and severe left ventricular systolic disfunction (Fig. 4).

Venous ultrasound of the lower limbs was normal.

**Fig. 5:**
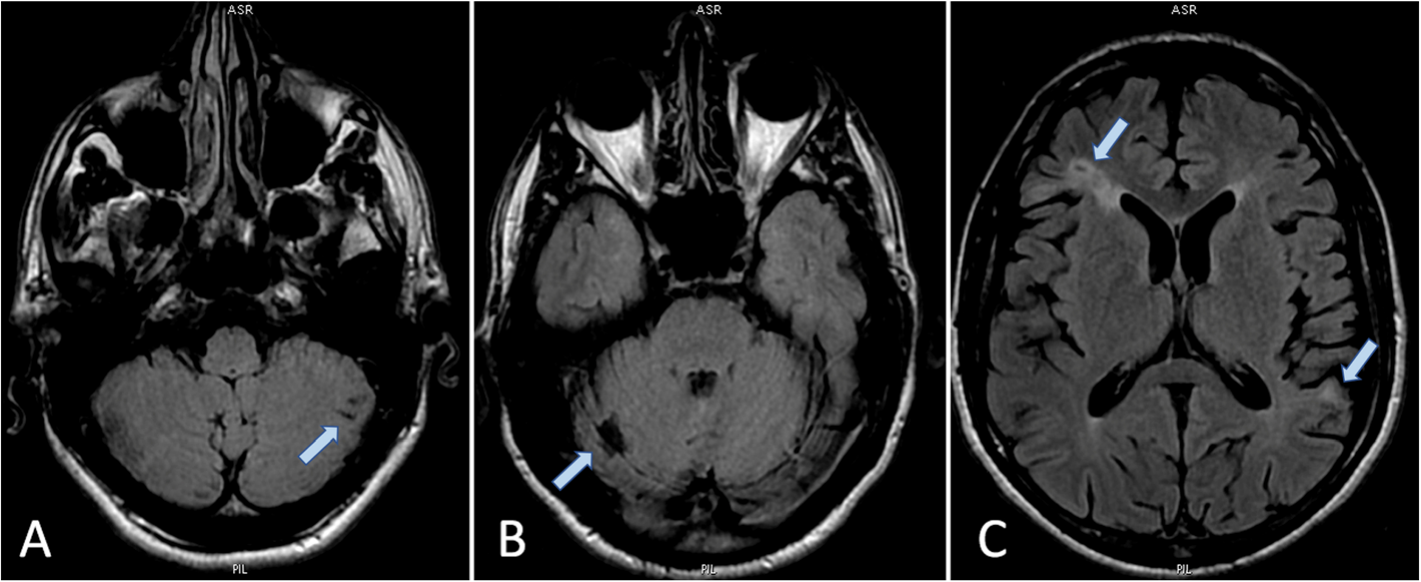
Brain T2-FLAIR MRI scans showing multiple areas of ischemia (*light blue arrows*) in the cerebellar and cerebral hemispheres. **a** Recent ischemic lesions in the lower left cerebellar hemisphere. **b** Old ischemic lesion in the upper right cerebellar hemisphere. **c** Recent ischemic lesions in the right frontal and left temporo-occipital cerebral regions

Because of the recent episode of dizziness and orthostatic imbalance, following neurologist review, a brain MR was also performed, documenting multiple areas of altered signal due to previous old and recent ischemic events in the cerebellar and cerebral hemispheres (Fig. 5).

Takayasu arteritis, giant cell arteritis and rheumatic diseases were considered in the differential diagnosis [4]; a rheumatologic screening was performed, including complement components, rheumatoid factor, anti-nuclear antibodies, anti-neutrophil cytoplasmic antibodies, anti-cardiolipin antibodies and β2-glycoprotein 1 antibodies, all of which resulted within the range of normality.

A thrombophilic screening was performed, including tests for protein C and protein S deficiencies, factor V Leiden and prothrombin 20,210 mutations, antithrombin deficiency, hyperomocysteinemia and antiphospholipid syndrome, and resulted negative.

The presence of mild pectus excavatum along with a history of surgery for foot deformity raised suspicion for collagen diseases such as Marfan and Ehlers-Danlos syndrome [4]; the genetic experts, however, due to the lack of significant physical signs of collagen disease and the absence of aortic root aneurysm and ectopia lentis [5], considered these diagnoses unlikely and genetic testing was not recommended.

From a cardiovascular point of view, two theories were hypothesized. According to the first one, the patient had suffered from a silent recent type 1 myocardial infarction leading to LVEF reduction with consequent thrombus formation in the left ventricle and subsequent embolization to the brain causing multi-infarct encephalopathy [6]; in this scenario, the PFO would be a mere bystander. The second theory infered that the patient had suffered from deep vein thrombosis from unknown origin, causing encephalic and myocardial infarction on embolic basis due to the right-to-left shunt of the PFO; in this latter case, the atherosclerotic LAD stenosis would be an incidental finding. Unfortunately, whatever the main event was, either the recent myocardial infarction or the deep vein thrombosis, the question remained unsolved due to its silent course.

**Fig. 6:**
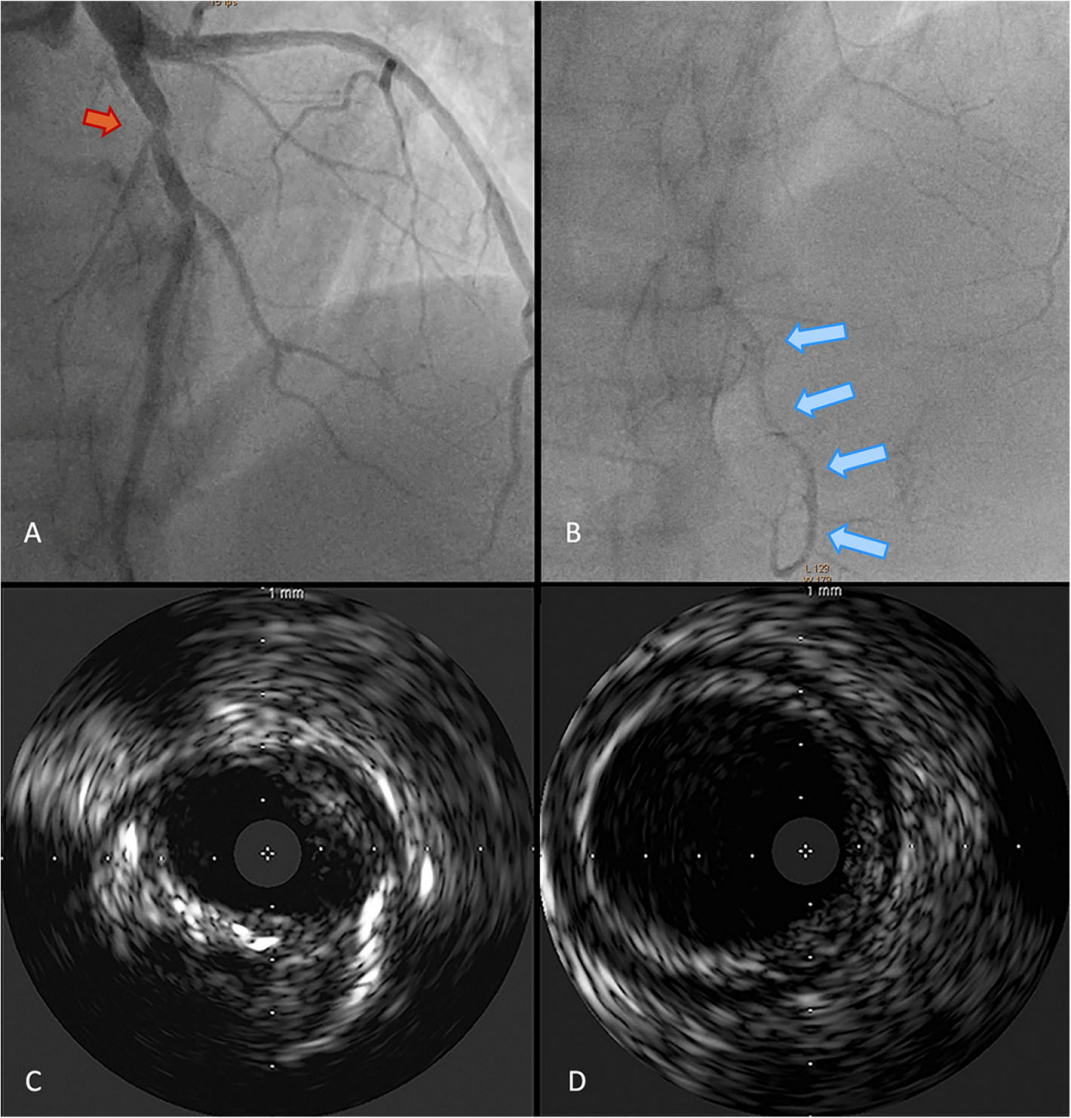
**a** Coronary angiography view showing 50% stenosis (*orange arrow*) of the proximal LAD coronary artery. **b** Coronary angiography view showing a long type 3 LAD coronary artery (*light blue arrows*) with patency of its distal portion. **c** IVUS image showing a minimal lumen area of 4.0 cm^2^ with high plaque burden (80%). **d** IVUS image showing patency of the segment adjacent to the LAD coronary artery stenosis

Following discussion between the cardiologist, the interventional radiologist and the cardiac surgeon, after multiple interviews with the patients regarding risks and benefits of the different treatment options, it was jointly decided not to perform endovascular treatment regarding the aortic dissection due to the lack of symptoms and the absence of parenchymal involvement, but careful monitoring was deemed necessary and strenuous exercise was contraindicated [2]. A coronary angiography exam was then performed showing 50% stenosis of the proximal LAD coronary artery at angiographic estimation (Fig. 6a) with patency of its distal segment (Fig. 6b); a “minus” image along with slow-flow appearance distal to the lesion raised suspicion for coronary artery dissection and induced the operator to evaluate the stenosis with intravascular ultrasound imaging, which revealed a critical minimal lumen area of 4,0 mm^2^ with 80% plaque burden (Fig. 6c) as compared to the patent lumen of the adjacent segment (Fig. 6d). Due to the high-risk features of the coronary lesion, after communicating benefit and risk information to the patient and acquiring informed consent, the operator performed angioplasty with the deployment of a Synergy™ drug-eluting stent. The patient was started on triple antithrombotic therapy with warfarin (because of the left ventricular thrombus), acetyl-salicylic acid and clopidogrel; low-molecular-weight heparin bridging was utilized until target INR (2.0–2.5) was achieved. The PFO closure procedure was temporarily postponed [7].

During the hospital stay the patient remained asymptomatic. Cardiac anti-remodelling therapy with angiotensin converting enzyme inhibitor and a potassium sparing diuretic was started and it was well tolerated. No arrhythmic events were documented on telemetry. At discharge the patient was asymptomatic and in stable haemodynamic condition.

**Fig. 7:**
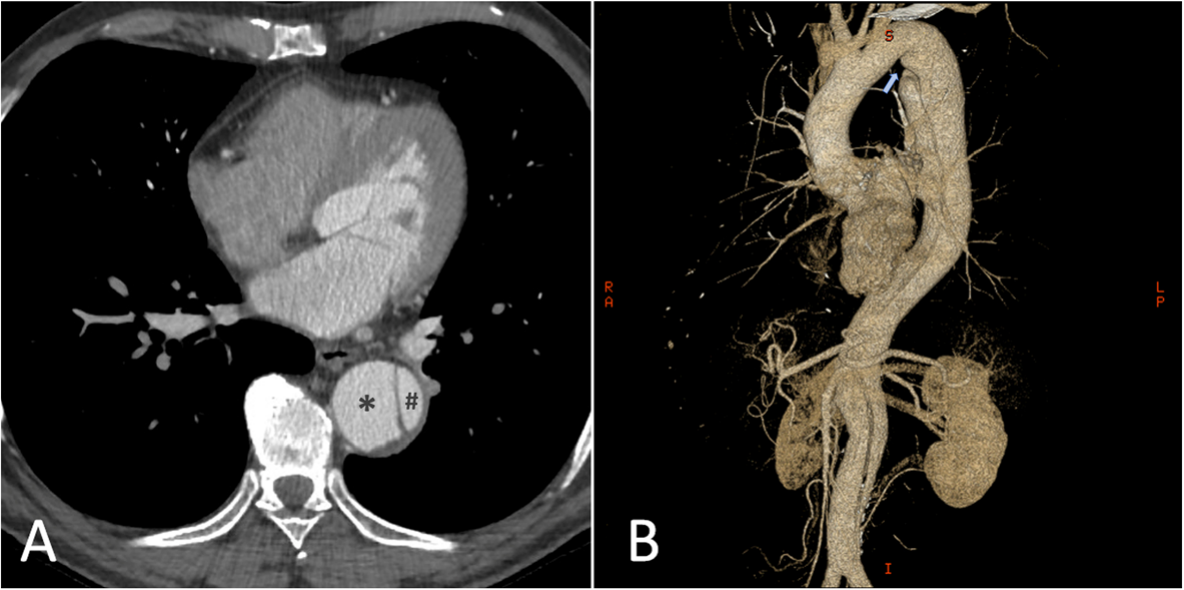
CTA image obtained 6 months after the acute event showing no progression of the aortic dissection. **a** Axial view showing the false lumen (*asterisk*) still prevailing over the true lumen (*hash*). **b** CTA 3D-scan showing the stability of the TBAD; the light blue arrow indicates the origin of the dissection

**Fig. 8:**
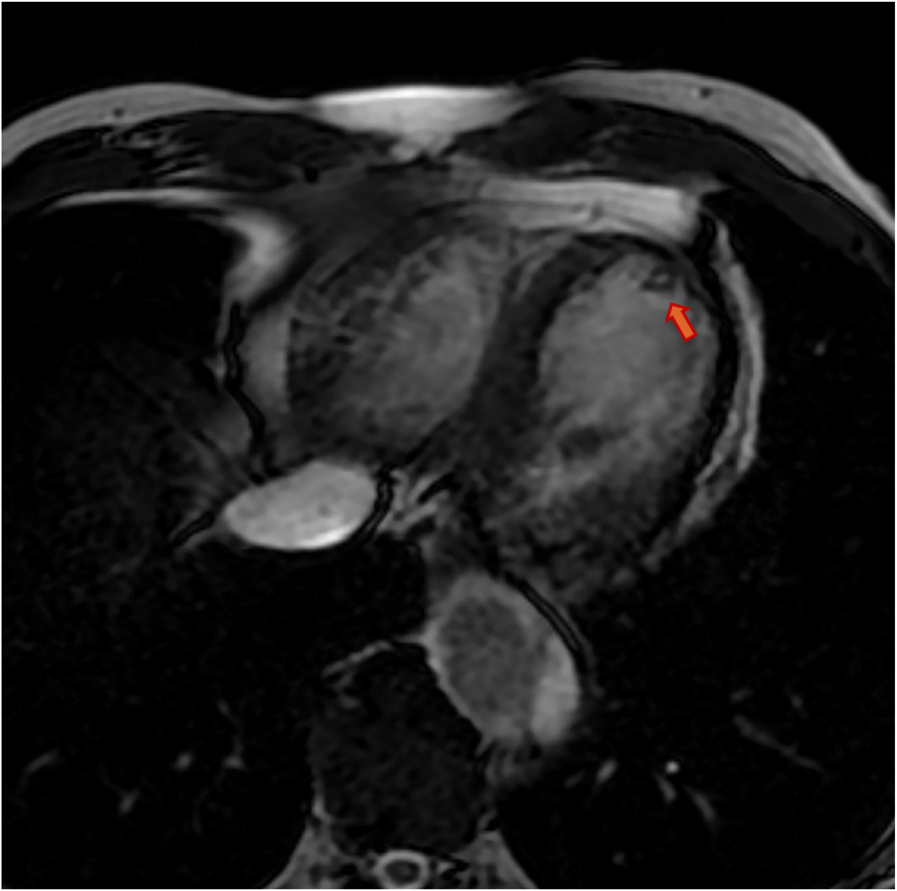
Early-enhancement cardiac MR image acquired 6 months after the acute event showing the presence of a tiny apical stratified thrombus (*orange arrow*) in the four-chamber view

After 1 month acetyl-salicylic acid was interrupted. After 6 months the CTA showed no progression of the aortic dissection (Fig. 7); LVEF was almost unchanged (38%) on TTE and a small intraventricular thrombotic formation, albeit reduced in diameter, still persisted on cardiac MR imaging (Fig. 8). The patient was asymptomatic and he was advised to prolong his therapy with both clopidogrel and warfarin until future assessment with TTE and CTA after 12 months. PFO closure was not deemed necessary as long as the patient was on anticoagulation therapy.

## Discussion and conclusion

Aortic dissection consists of a tear in the intimal layer of the aortic wall, thus connecting the media with the aortic lumen and allowing the blood to flow from the true lumen, delimited by the intimal layer, into the false lumen, localized between the intima and media of the aortic wall [2]. High pressure within the aortic wall causes the rupture to propagate either anterogradely or retrogradely or both and can lead to hypoperfusion of parenchymal organs and increasing risk of aortic rupture [8].

According to Stanford’s classification, aortic dissections are classified as type A when they involve the ascending aorta and as type B if only the descending aorta is involved [9]. TBAD can further be subdivided into complicated or uncomplicated depending on the presence of at least one of the following: malperfusion syndrome, aortic rupture, hypotension or shock, neurologic sequelae, recurrent or refractory pain, hypertension refractory to medical therapy, early aortic dilation or propagation of the dissection [2]. The first-line treatment of acute TBAD is “anti-impulse” therapy, which consists of aggressive control of blood pressure. With regard to the use and management of anticoagulants in patients with TBAD, recommendations are completely missing in current guidelines [2].

A study conducted by Akutsu et al. in 2004 showed that patency of the false lumen is a strong independent prognostic factor for dissection-related death in patients with TBAD [10]. More recent reports, however, have questioned this issue; Tsai et al. in 2007 showed that no difference in mortality exists between TBAD patients with complete thrombosis of the false lumen and patients with a completely patent false lumen, whereas partial thrombosis of the false lumen was associated with a significantly higher mortality as compared with patency of the false lumen (HR 2.69, 95% CI 1.45–4.98, *p*-value 0.002) [11]. Nevertheless, several case reports in literature demonstrate the suitability and apparent safety of anticoagulants in the setting of acute aortic syndrome [12, 13]. Cañadas et al. described the absence of adverse post-hospitalization outcomes in three patients with intramural aortic hematoma and history of pulmonary embolism or atrial fibrillation that were treated initially with unfractionated heparin and then with warfarin, concluding that in situations where anticoagulation is necessary this treatment can be maintained without an impact on the clinical or morphological evolution of the intramural aortic hematoma [14]. Bhatty et al. described a patient with chronic TBAD that suffered of pulmonary embolism and was treated with administration of intravenous heparin therapy and then discharged with warfarin therapy for 3 months without complications [15]. Von Kodolitsch et al. showed that anticoagulation with warfarin is feasible even in post-surgical patients with type A aortic dissection as it did not affect aortic growth and was not associated with late mortality nor with late aortic events, thus suggesting that patients requiring anticoagulation due to other indications should be indeed considered on individualized basis to receive such treatment [16]. This holds true especially for atrial fibrillation, which is not uncommon among patients with aortic dissection [17]; few reports have addressed the issue of anticoagulation in this scenario with heterogeneous results and patient-tailored therapy seems the best treatment option until larger studies will focus on this topic [18, 19].

Our case presents a rare association of pathologies requiring antithrombotic therapy in the context of acute TBAD. The necessity to treat the proximal LAD stenosis with the deployment of a drug-eluting stent in a patient with intraventricular thrombosis required triple antithrombotic treatment; after multidisciplinary assessment, the risk of thromboembolism from the left ventricle and the necessity to treat the coronary lesion were considered to outweigh the potential risk of dissection progression and aortic rupture given by false lumen patency [20]. The aortic dissection was chosen not to be repaired due to the uncomplicated course of the disease at this point. PFO closure was not considered a priority anymore due to the protection from further strokes granted by anticoagulant agents and because the use of a device for this procedure would prolong the duration of antithrombotic treatment [7].

The evidence of a patent false lumen and the uncomplicated course of the aortic dissection after 6 months of antithrombotic therapy show that, along with previous reports [11–14], anticoagulation should not be considered an absolute contraindication in every patient with TBAD but needs to be evaluated on a case-by-case basis. To our knowledge no large randomized trial is being conducted to face this relevant issue, thus this case supports the necessity of a multi-disciplinal consensus on the management of anticoagulation therapy in TBAD patients, which is a controversial matter often taken for granted. We believe that the acute setting must be faced with caution and the benefits of anticoagulation therapy must be carefully balanced against the potential necessity for surgical intervention due to dissection progression, which is difficult to estimate in the first hours, or severe parenchymal suffering. Markers of parenchymal organs damage must be frequently assessed and anticoagulation therapy might be withheld in the hyperacute setting until it is decided whether to medically or surgically treat the patient. When concomitant conditions require antithrombotic therapy and the risk of thrombosis exceeds the risk of bleeding, short-acting anticoagulants might be preferred in the acute setting due to their shorter half-life and easier handling, should unpredictable conditions requiring anticoagulation interruption arise; moreover, fast-acting anticoagulant agents, such as unfractionated or low-molecular-weight heparin, could provide a bridge to oral vitamin K antagonists, which usually require a few days before adequate antithrombotic status is achieved. Heparin bridging proved safe and feasible in our case, but no absolute recommendation can be given due to the absence of targeted studies. At discharge oral anticoagulation therapy should be preferred over heparin-based drugs, due to its safety and easier route of administration. As many different conditions may require the use of anticoagulation therapy, no absolute statement can be given as for the duration of this therapy; reversible thrombotic conditions might require shorter anticoagulation duration, whereas irreversible conditions, such as permanent atrial fibrillation, might require indefinite anticoagulation. Randomized studies regarding the use of novel oral anticoagulants in patients with aortic dissection are lacking; for this reason, no recommendation can be given about which type of anticoagulant agent should be preferred. A case report by Turgay Yıldırım et al., however, shows that dabigatran might be safe in the setting of TBAD and we believe that novel oral anticoagulants might be used in this scenario as well [19]. Nevertheless, studies are needed to specifically address this issue. As the aortic dissection becomes chronic, periodic imaging examinations must be performed in order to assess the stability and extension of the dissection and false lumen patency and anticoagulation therapy should be managed accordingly after ischemic and bleeding risk assessment.

In conclusion, despite it is generally considered unsafe to administer anticoagulants in patients with type B aortic dissection, very few studies focused on this subject and different reports state that anticoagulant therapy might be harmless or might even improve the prognosis of this set of patients. Each TBAD patient requiring anticoagulation should be approached individually, carefully balancing ischemic and bleeding risks, which should be extensively discussed with the patient; a personalized assessment and multidisciplinary decision-making is a key issue in this regard. However, there is a large evidence gap and uncertainty in this context and trials in this arena are urgently needed.

## Data Availability

The datasets used and/or analysed during the current study are available from the corresponding author on reasonable request.

## Abbreviations

LAD: Left anterior descending artery
LV: Left ventricular
PFO: Patent foramen ovale
TBAD: Type B aortic dissection
